# Exploring the Role of Meditation and Dispositional Mindfulness on Social Cognition Domains: A Controlled Study

**DOI:** 10.3389/fpsyg.2019.00809

**Published:** 2019-04-11

**Authors:** Daniel Campos, Marta Modrego-Alarcón, Yolanda López-del-Hoyo, Manuel González-Panzano, William Van Gordon, Edo Shonin, Mayte Navarro-Gil, Javier García-Campayo

**Affiliations:** ^1^Laboratorio de Psicología y Tecnología, Department of Basic Psychology, Universitat Jaume I, Castelló de La Plana, Spain; ^2^Instituto de Investigación Sanitaria de Aragón, IIS Aragón, Zaragoza, Spain; ^3^Department of Medicine, Psychiatry and Dermatology, University of Zaragoza, Zaragoza, Spain; ^4^Primary Care Prevention and Health Promotion Research Network, Madrid, Spain; ^5^Human Sciences Research Centre, University of Derby, Derby, United Kingdom; ^6^Awake to Wisdom Centre for Meditation and Mindfulness Research, Ragusa, Italy; ^7^University Hospital Miguel Servet, Zaragoza, Spain

**Keywords:** mindfulness, meditation, social cognition, empathy, emotional recognition, theory of mind

## Abstract

Research suggests that mindfulness can induce changes in the social domain, such as enhancing emotional connection to others, prosocial behavior, and empathy. However, despite growing interest in mindfulness in social psychology, very little is known about the effects of mindfulness on social cognition. Consequently, the aim of this study was to explore the relationship between mindfulness and social cognition by comparing meditators with non-meditators on several social cognition measures. A total of 60 participants (meditators, *n* = 30; non-meditators, *n* = 30) were matched on sex, age, and ethnic group, and then asked to complete the following assessment measures: Mindful Awareness Attention Scale (MAAS), Five Facet Mindfulness Questionnaire Short Form (FFMQ-SF), Interpersonal Reactivity Index (IRI), Revised Eyes Test, Hinting Task, Ambiguous Intentions and Hostility Questionnaire (AIHQ), Hospital Anxiety and Depression Scale (HADS), and Screening for Cognitive Impairment in Psychiatry (SCIP). The results showed that meditators reported higher empathy (except for the personal distress subscale), higher emotional recognition, higher theory of mind (ToM), and lower hostile attributional style/bias. The findings also demonstrated that dispositional mindfulness (both total score assessed with MAAS and mindfulness facets using the FFMQ) was associated with social cognition, although it was not equally correlated with all social cognition outcomes, and correlation patterns differ when analyses were conducted separately for meditators and non-meditators. In addition, results showed potential predictors for each social cognition variable, highlighting non-reactivity to inner experience as a key component of mindfulness in order to explain social cognition performance. In summary, the findings indicated that the meditator sample performed better on certain qualities (i.e., empathy, emotional recognition, ToM, hostile attributional style/bias) in comparison to non-meditators and, furthermore, support the notion that mindfulness is related to social cognition, which may have implications for the design of mindfulness-based approaches for use in clinical and non-clinical settings.

## Introduction

Research into mindfulness has increased over the last decade and, consequently, several interventions to promote mindfulness skills have been implemented and widely used in clinical settings (Khoury et al., 2013; Demarzo et al., 2015; Van Gordon et al., 2017). Mindfulness has been conceptualized by Western culture in several ways, and reference is currently made to dispositional (or trait) mindfulness, state mindfulness, and to the actual techniques of mindfulness training (e.g., mindfulness meditation) (Grossenbacher and Quaglia, 2017). Formal meditation, understood as psychological or mind training, has shown to be useful to enhance both dispositional and state mindfulness (e.g., Baer, 2003; Baer et al., 2008, 2012; Kiken et al., 2015), although mindfulness can also be trained through informal practice, such as mindfulness in daily life activities (Salmon et al., 1998; Carmody and Baer, 2008). There is a growing body of evidence highlighting the benefits of mindfulness on health, well-being, attention, cognitive functioning, and cognitive flexibility (e.g., Moore and Malinowski, 2009; Van Gordon et al., 2014; Khoury et al., 2015; Campos et al., 2016; Mira et al., 2016; Raffone and Srinivasan, 2017). Furthermore, mindfulness training has been used to elicit changes in self-awareness, emotion regulation, and neurophysiology (e.g., Ospina et al., 2007; Slagter et al., 2007; Lutz et al., 2008; Chiesa et al., 2011; Vago and Silbersweig, 2012; Lippelt et al., 2014). Nevertheless, given that one of the foundations of mindfulness is to enhance compassionate thoughts, feelings, and behaviors (Dalai Lama, 1995; Wallace, 2001), another important area of interest is whether mindfulness elicits changes in the social domain. Specifically, little is known about how mindfulness is related to social cognition. In this regard, some authors have suggested that meditation and mindfulness skills could be useful tools to promote social cognition domains, although research in this field is scarce, and few studies have formally addressed such issues.

### Social Cognition (SC)

Social cognition can be defined as “the mental operations that underlie social interactions, including perceiving, interpreting, and generating responses to the intentions, dispositions, and behaviors of others” (Green et al., 2008, p. 1211). It involves the capacity to understand oneself and others (Lieberman, 2007), and comprises four core domains of social cognition including (i) emotion processing, (ii) social perception, (iii) theory of mind/mental state attribution (ToM), and (iv) attributional style/bias (Pinkham et al., 2014). ToM refers to a person’s capacity to attribute mental states (e.g., intentions, beliefs, desires, etc.) to both themselves and others, and to appreciate that others can have mental states that differ from their own (Corcoran et al., 1995).

Another construct that has been widely related to social cognition is empathy, which implies a knowledge of others that is more embodied than logical, and which requires the individual to maintain an awareness that the emotional response is an embodied simulation of another person’s experience, not to be confused with one’s own experience (Lieberman, 2007). Overall, studies have demonstrated the association between deficits across social cognition domains and psychopathology, highlighting their implications for mental health (Barbato et al., 2015; Gallagher and Varga, 2015; Lahera et al., 2015).

### Effects of Mindfulness Meditation (and Dispositional Mindfulness) on Social Cognition Domains

Most of the extant empirical literature investigates the role of meditation practice and mindfulness (both state and trait) in the field of emotional processing, which is broadly defined as perceiving and using emotions, including recognition perception, understanding and managing emotions (Green et al., 2008; Pinkham et al., 2014). For example, a brief (20-min) guided mindfulness meditation exercise led to significant changes in emotional processing indicative of reduced emotional reactivity (Lin et al., 2016). The study authors reported that these effects were akin to those observed in individuals with naturally high dispositional mindfulness, suggesting that the benefits of mindfulness can be cultivated through practice. Another study showed that after a brief mindfulness induction condition (15-min recording), participants were more mindful of how they focused their attention and were able to better regulate their response to the negative images (Eddy et al., 2015). A further study showed the influence of long-term meditation practice on early emotional processing in the brain, indicating that long-term meditation practice enhances frontal top-down control over fast automatic detection of stimulus salience (Reva et al., 2015).

Additionally, there evidence in respect of the impact of mindfulness on empathy (e.g., Birnie et al., 2010; Raab, 2014; Jones et al., 2016). More specifically, increased attention has been shown to enhance empathy, together with compassion and prosocial behavior, indicating small-to-medium effects of meditation based on a systematic review and meta-analysis (Luberto et al., 2018). Furthermore, Ridderinkhof et al. (2017) investigated whether mindfulness meditation (5-min exercise) increased empathy, and they observed no effect of mindfulness relative to both control conditions (relaxation and mind-wandering) on mind reading (ToM), empathic responding, or prosocial behavior. The authors also found that mindfulness meditation improved mind reading (i.e., ToM) only in non-narcissistic people, questioning whether a brief mindfulness exercise would be sufficient for building empathy.

Another study revealed that a brief mindfulness meditation (5-min mindfulness induction) enhanced both ToM and empathic concern, compared with the control group (Tan et al., 2014). Findings from the study, together with studies suggesting linkages between ToM and mindfulness (i.e., improved executive attention, improved cognitive operations to map the minds of self and others via present-moment thinking, overlapping of cortical regions that play a role during meditation and for mindreading and self-referential mental activity; e.g., Farb et al., 2007; Siegel, 2007; Tang et al., 2009; Hölzel et al., 2011a), support the idea that mindfulness meditation may play a powerful role in promoting core aspects of social cognition functions.

Furthermore, Melloni et al. (2013) investigated the comprehensive effects of meditation on several domains of social cognition such as emotional recognition, empathy and ToM facets of social cognition. The results of their study indicated that meditators’ performance did not differ when compared with that of non-meditators (controls), except on reported lower accuracy in disgust emotion recognition in long-term meditators and lower personal distress (empathy subscale) in both long- and short-term meditators (i.e., versus non-meditators). However, while these findings were asynchronous with extant theory and the consensual understanding of mindfulness and social cognition, the study sample only comprised 10 non-meditators, 10 long-term meditators, and 9 short-term meditators. Therefore, further research is clearly needed in order to gauge whether these outcomes are robust.

### Social Mindfulness

Despite studies claiming a role for meditation and dispositional mindfulness in social cognition domains, there is a noteworthy gap in the literature with regard to how meditation and dispositional mindfulness (including mindfulness facets) are associated with specific social cognition domains - i.e., how paying attention to the experience of the present moment affects the way people see and interact with the world. Thus, there is a lack for theoretical background in order to build a solid conceptual model.

An interesting proposal is suggested by Van Doesum et al. (2013), who adopted the novel concept of social mindfulness and conducted a series of studies suggesting how social mindfulness can help people to navigate the social world based on an interdependence and decision-making theoretical approach. According to these authors, social mindfulness is minding the needs and interests of others in a way that honors the idea that most people like to choose for themselves. Thus, it is conceptualized in terms of other-regarding choices involving both skill (to see it, e.g., ToM, perspective-taking) and will (to do it, e.g., empathic concern, prosocial orientation) to act mindfully toward another person’s control over outcomes. Several of Van Doesum et al.’s (2013) study findings are worth highlighting: (i) people with other-oriented mindsets left interdependent others more choice than people with self-oriented and/or unspecified mindsets; (ii) people developed more favorable judgments of a socially mindful versus a socially unmindful person; (iii) unknown others with trustworthy (vs. untrustworthy) faces were met with more social mindfulness; (iv) social mindfulness could be traced in personality by being positively related to Honesty-Humility and Agreeableness, Empathy, and a prosocial value orientation. Findings from this study warrant the importance of further research on this issue, focusing on mindfulness and social cognition in order to better understand how these constructs are related.

### Aims of the Study

The present authors propose that there exists a significant relationship between meditation practice, dispositional mindfulness and performance across social cognition domains. More specifically, we propose that meditation practice cultivates mindfulness skills (i.e., awareness of internal and external experiences by broadening perspective without automatically reacting) which implies a greater ability to perceive, interpret, and generate responses to the intentions, dispositions, and behaviors of others (i.e., the core of social cognition domains). A study confirming our assumptions would imply that mindfulness can be a useful technique for modifying how a person perceives the world by enhancing their performance on social cognition variables such as emotion recognition, ToM, attributional style/bias and empathy. More generally, identifying how specific facets of mindfulness relate to social cognition domains should foster a better comprehension of the social mind and behavior and, thus, help to promote wellbeing in the general population as well as improve psychological interventions focused on social cognition deficits.

Accordingly, the aim of the present study was to explore the role of meditation and dispositional mindfulness on social cognition. First, we aimed to compare the performance of meditators and non-meditators on a battery of social cognition measures. Second, we aimed to explore the relationship between dispositional mindfulness and social cognition measures. Third, we also focused on exploring whether dispositional mindfulness (and which specific facets of mindfulness) significantly predict social cognition outcomes. It was hypothesized that the meditation group would perform better on indices of social cognition performance (i.e., empathy, emotional recognition, ToM, and hostile attributional style/bias) versus non-meditators. Furthermore, it was hypothesized that dispositional mindfulness (overall score and mindfulness facets) would be associated with social cognition (empathy, emotional recognition, ToM, and attributional style) and significantly explain the variance of social cognition outcomes.

## Materials and Methods

### Design

**FIGURE 1:**
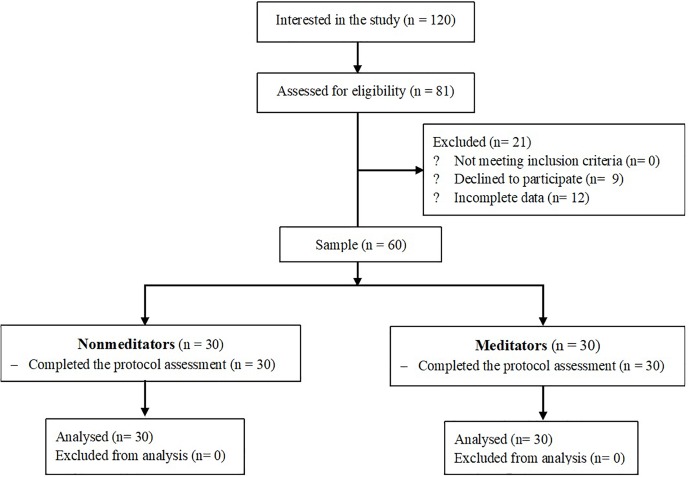
Flow chart of the study.

A cross-sectional two-arm design was used. The study followed Helsinki Convention norms and posterior modifications, and the Declaration of Madrid of the World Psychiatric Association. The Clinical Research Ethics Committee of Aragon approved the study protocol. Figure 1 shows the flow chart.

### Participants

A total of 60 participants (meditators, *n* = 30; non-meditators, *n* = 30) were enrolled in the study. The meditation group comprised students from a Masters in Mindfulness Program at the University of Zaragoza, Spain. The non-meditation group (controls) comprised healthy volunteers from the community without meditation experience. The mean age was 42.50 (*SD* = 7.83) and ranged between 26 and 55 years (male, *n* = 20; female, *n* = 40). Most participants had completed higher education (73.3%), were in fulltime work (73.3%), and married (60%). Using visual inspection of demographic information, the non-meditators were matched with meditators on sex, age, and ethnicity.

### Recruitment and Procedure

The study was advertised on the webpage of the Masters in Mindfulness of the University of Zaragoza. To be eligible for the meditation group, participants had to have at least 1 year’s meditation experience prior to the start of the study. Additional eligibility criteria (for both the meditation and non-meditation group) were (i) an age of between 18 and 65 years, (ii) ability to read and understand Spanish, and (iii) not having been diagnosed with a mental disorder or undergoing psychiatric pharmacological treatment. Participants in the meditation group were advised not to meditate for 2 h prior to or while undergoing the assessment. Informed consent was obtained from all participants.

### Measures

#### Socio-Demographic and Meditation Data

Socio-demographic data were obtained for age, sex, occupation, educational attainment, and presence of a psychiatric condition or psychological disorder. Mindfulness practice data were assessed using a brief questionnaire (Soler et al., 2014a; Campos et al., 2016; Cebolla et al., 2017) containing questions relating to meditation experience (yes/no), frequency of meditation practice (every day, three or four times a week, once a week, two or three times per month, or sporadically), years of meditation experience, and the average duration of each meditation session in minutes.

#### Dispositional Mindfulness Measures

The *Mindful Attention Awareness Scale* (MAAS) is a 15-item scale that assesses the individual’s dispositional capacity to be aware and conscious during every moment of the day (Brown and Ryan, 2003; Soler et al., 2012). A Spanish version of the scale – demonstrated to have good psychometric properties (Cronbach’s α = 0.897) – was used in the current study (Soler et al., 2012). The Cronbach’s alpha value for the sample in this study was 0.91.

The *Five Facet Mindfulness Questionnaire* – *Short form* (FFMQ-SF) (Bohlmeijer et al., 2011) is a 24-item short-form version of the FFMQ (Baer et al., 2006, 2008; Cebolla et al., 2012; Aguado et al., 2015) that assesses five different facets of mindfulness: (i) *observing*, which refers to the individual’s capacity to pay attention to internal and external experiences such as sensations, thoughts, and emotions, (ii) *describing*, which assesses the ability to describe events and personal responses in words, (iii) *acting with awareness*, which involves focusing on the activity being carried out instead of behaving automatically, (iv) *non-judging of inner experience*, which refers to the ability to take a non-evaluative stance toward thoughts and feelings, and (v) *non-reactivity to inner experience*, which involves allowing thoughts and feelings to come and go without getting caught up in or carried away by them (Baer et al., 2008). Both the FFMQ and FFMQ-SF, as well as the validated Spanish version, have shown to be reliable and valid instruments in adult populations. The Cronbach’s alpha value for the sample in this study was 0.86 for the total score and ranged between 0.63 and 0.76 for the subscales (observe, α = 0.73; describe, α = 0.63; awareness, α = 0.66; non-judging, α = 0.76; non-reactivity, α = 0.66).

#### Social Cognition Measures

##### Empathy

The *Interpersonal Reactivity Index* (IRI) (Davis, 1980, 1983) is a 28-item questionnaire scored on a Likert scale ranging between 0 (“doesn’t describe me at all”) and 4 (“describes me very well”). The IRI assesses four components of empathy including (i) fantasy (F) (i.e., the tendency to identify with fictitious characters), (ii) perspective-taking (PT), (iii) empathic concern (EC), and (iv) personal distress (PD) in the face of others’ suffering. The IRI has been validated in Spanish with psychometric properties similar to those of the original English-language version (Pérez-Albéniz et al., 2003). For the sample in this study, the alpha value ranged between 0.52 and 0.75 for the subscales (fantasy, α = 0.52; perspective-taking, α = 0.75; empathic concern, α = 0.73; personal distress, α = 0.58).

##### Emotion recognition

The *Eyes Test* (revised version) is a measure comprising 36 photographs of the eye region of the faces of different actors and actresses. Respondents are asked to choose which of four words best describes what the person in the photograph is thinking or feeling (Baron-Cohen et al., 2001). The test has proven to be a sensitive measure of adult social intelligence (Baron-Cohen et al., 2001, 2015). The Spanish version, used in this study, has shown that the Eyes Test is reliable and stable over a 1-year period, in a non-clinical sample of adults (Fernández-Abascal et al., 2013). For the sample in this study, the alpha value was 0.62.

##### Theory of mind (ToM)

The *Hinting Task* was developed to assess the ability of respondents to infer the true intention behind indirect speech utterances throughout 10 short passages reflecting an interaction between two characters (Corcoran et al., 1995). The total score range is 0–20. A detailed description of the task, instructions and correction form can be found in Corcoran et al. (1995). The Hinting Task has demonstrated good psychometric properties in the validated Spanish version (Corcoran et al., 1995; Greig et al., 2004; Gil et al., 2012). For the sample in this study, the alpha value was 0.57.

##### Attributional style

The *Ambiguous Intentions and Hostility Questionnaire* (AIHQ) is a measure to assess hostile social-cognitive biases (Combs et al., 2007). This task is comprised of 15 short vignettes that reflect negative outcomes that vary in intentionality (i.e., intentional, accidental, and ambiguous intention). Respondents are asked to read each vignette, imagine the scenario happening to them, and to write down (i) the reason why the other person or persons acted in a particular manner (Hostility Bias, AIHQ-HB subscale), (ii) whether the other person or persons performed the action on purpose (Intentionality Bias, AIHQ-IS subscale), (iii) how angry it made them (the respondents) feel (Anger Bias, AIHQ-AS subscale), (iv) how much they would blame the other person or persons (Blame Scale, AIHQ-BS subscale), and (v) how they would respond to the situation (Aggressivity Bias, AIHQ-AB subscale). The AIHQ had good internal consistency and interrater reliability (Combs et al., 2007). For the sample in this study, the alpha values ranged between 0.81 and 0.91 for the subscales (Hostility Bias, α = 0.91; Intentionality Bias, α = 0.77; Anger Bias, α = 0.86; Blame Scale, α = 0.84; Aggressivity Bias; α = 0.81).

#### Other Measures

##### Depression and anxiety

The *Hospital Anxiety and Depression Scale* (HADS) (Zigmond and Snaith, 1983; Crawford et al., 2001) is a self-report tool that contains 14 items measured on a four-point Likert scale that assess anxiety and depression. The Spanish version of HADS has shown good psychometric properties for both psychiatric and healthy samples (Tejero et al., 1986; Terol et al., 2007). For the sample in this study, the alpha value was 0.79.

##### Cognitive impairment

The Screen for Cognitive Impairment in Psychiatry (SCIP; Purdon, 2005) is a brief scale to assess cognitive impairment, including immediate and delayed verbal learning, working memory, verbal fluency, and psychomotor speed. The validated Spanish version of the SCIP has demonstrated properties similar to those found in the English version (Pino et al., 2006). For the sample in this study, the alpha value was 0.74.

### Data Analyses

Differences between groups regarding socio-demographic data were assessed using chi-square (χ^2^) tests for categorical variables (sex, educational attainment, and occupation) and *t*-tests for continuous variables (age). Independent Student *t*-tests were used to assess mean differences between meditators and non-meditators on each study measure. Between-group effect sizes (Cohen’s *d*; 95% CI) were also calculated and reported (Cohen, 1988; Lakens, 2013; Botella and Sánchez-Meca, 2015). Separate analyses of covariance (ANCOVA) or multivariate analyses of covariance (MANCOVA) (when appropriate) controlling for socio-demographic differences (educational attainment) were added on each dependent variable (VD) in order to explore the influence of significant demographic data. Pearson correlations were conducted to explore the relationship between dispositional mindfulness and social cognition measures for the total sample, and separately for both meditator and non-meditator samples. Finally, multiple linear regressions (stepwise method) were carried out to examine the dispositional mindfulness variables that predict social cognition domains. Thus, dispositional mindfulness (assessed by MAAS), mindfulness facets, and educational attainment were entered simultaneously in order to determine which factors significantly contributed to explaining the variance in each domain of social cognition. All analyses were based on completers and performed using IBM SPSS Statistics 23.0 for Windows.

## Results

Table 1 shows the socio-demographic data for each group separately. No statistically significant differences between groups were identified in socio-demographic characteristics, except for educational attainment [X^2^(2) = 9.318; *p* < 0.01], where the meditators group showed a higher proportion of secondary and university students. In terms of frequency of practice, 13.3% of meditators reported practicing formal meditation every day; 43.3% practiced three or four times a week; 20% practiced once a week; 6.7% practiced two or three times per month; and 16.6% meditated sporadically. The mean years of meditation experience was 3.57 (*SD* = 7.46). On average, participants meditated for 18.67 min (*SD* = 12.80) per session.

**Table 1:** Socio-demographic data for each group (meditators and non-meditators).

	Meditators *n* = 30	Non-meditators *n* = 30	Statistics
Age			
Mean (SD) [range]	42.56 (7.5) [27–55]	42.43 (8.27) [26–55]	*F*_(1,59)_ = 0.004 *p* = 0.948
Gender, *n* (%)			
Male	10 (33.3%)	10 (33.3%)	*X*^2^_(1)_ = 0.00
Female	20 (66.7%)	20 (66.7%)	*p* = 1.00
Education, *n* (%)			
Elementary	0 (0.0%)	8 (26.7%)	*X*^2^_(2)_ = 9.318
Secondary	5 (16.7%)	3 (10.0%)	*p* < 0.01
University	25 (83.3%)	19 (63.3%)	
Marital status *n* (%)			
Single	9 (30.0%)	8 (26.7%)	*X*^2^_(6)_ = 5.503
Married	20 (66.7%)	16 (53.3%)	*p* = 0.138
Divorced	0 (0.0%)	5 (16.7%)	
Widowed	1 (3.3%)	1 (3.3%)	
Employment			
Unemployed	10 (33.3%)	2 (6.6%)	*X*^2^_(2)_ = 10.891
Employed	6 (20.0%)	23 (76.7%)	*p* = 0.054
Retired	0 (0.0%)	4 (13.3%)	
Disability	14 (46.7%)	1 (3.3%)	


*SD, standard deviation. Disability refers to employees with short-term disability (sick leave) at the time that the study was conducted.*

Means comparisons showed statistically significant differences between meditators and non-meditators for social cognition measures with effect sizes ranging between 0.60 and 1.22 (Table 2). Meditators scored significantly higher on *empathy* subscales: *Fantasy* [*t*(58) = -2.35; *p* < 0.05], *perspective-taking* [*t*(58) = -4.773; *p* < 0.001], and *empathic concern* [*t*(58) = -3.739; *p* < 0.001]. No significant differences were found in the *personal distress* subscale. In relation to *emotional recognition*, meditators scored significantly higher than non-meditators, as reflected by Eyes Test scores [*t*(58) = -2.370; *p* < 0.05]. Similarly, statistically significant differences were found for *ToM* [*t*(58) = -2.495; *p* < 0.05], where meditators demonstrated better performance in the Hinting Task versus non-meditators. For *attributional style*, meditators scored statistically lower than non-meditators, revealing lower *hostility bias* [*t*(58) = 2.784; *p* < 0.01], *intentionality bias* [*t*(58) = 4.361; *p* < 0.001], *blame* [*t*(58) = 3.989; *p* < 0.001], *anger bias* [*t*(58) = 4.515; *p* < 0.001], and *aggressivity bias* [*t*(58) = 2.358; *p* < 0.05].

**Table 2:** Comparisons between meditators and non-meditators on social cognition and dispositional mindfulness measures.

	Meditators (*n* = 30)	Non-meditators (*n* = 30)		
Measures	M (SD)	M (SD)	*t*	Cohen’s *d* [95% CI]
IRI				
IRI_PT	25.23 (1.63)	21.83 (3.54)	–4.773***	*d* = 1.22 [0.67, 1.77]
IRI_FS	21.07 (3.93)	18.80 (3.57)	–2.325*	*d* = 0.60 [0.08, 1.11]
IRI_EC	24.47 (4.35)	20.63 (3.55)	–3.739***	*d* = 0.95 [0.42, 1.49]
IRI_PD	16.40 (4.74)	17.73 (3.55)	1.234	*d* = –0.31 [–0.82, 0.20]
Eyes test	25.50 (3.12)	22.97 (4.96)	–2.370*	*d* = 0.60 [0.09, 1.12]
Hinting Task	17.53 (1.46)	16.03 (2.96)	–2.495*	*d* = 0.63 [0.12, 1.15]
AIHQ				
HB	15.43 (7.50)	21.97 (10.44)	2.784**	*d* = –0.71 [–1.23, –0.19]
IS	33.23 (9.73)	42.63 (6.69)	4.361***	*d* = –1.11 [–1.65, –0.57]
AS	31.37 (8.71)	40.67 (7.10)	4.515***	*d* = –1.16 [–1.70, –0.61]
BS	31.80 (9.67)	40.27 (6.46)	3.989***	*d* = –1.02 [–1.55, –0.48]
AB	23.93 (5.63)	28.63 (9.36)	2.358*	*d* = 0.60 [–1.12, –0.08]
MAAS	4.31 (.84)	3.53 (.91)	–3.463**	*d* = 0.88 [0.35, 1.41]
FFMQ				
*Observing*	16.07 (2.48)	12.93 (3.11)	–4.320***	*d* = 1.10 [0.28, 0.56]
*Describing*	16.30 (2.91)	14.57 (2.66)	–2.406*	*d* = 0.61 [0.09, 1.13]
*Awareness*	13.07 (3.76)	15.10 (3.50)	2.169*	*d* = –0.55 [–1.07, –0.04]
Non-judgment	10.10 (3.17)	12.17 (3.50)	2.370*	*d* = –0.61 [–1.13, –0.09]
Non-reactivity	19.37 (2.30)	15.63 (2.77)	–5.679*	*d* = 1.45 [0.88, 2.02]


*M, mean; SD, standard deviation; IRI, Interpersonal Reactivity Index; FS, fantasy; PT, perspective-taking; EC, empathic concern; PD, personal distress; AIHQ, Ambiguous Intentions and Hostility Questionnaire; HB, Hostility Bias; IS, Intentionality Bias; AS, Anger Bias; BS, Blame Scale; AB, Aggressivity Bias; MAAS, Mindful Attention Awareness Scale; FFMQ, Five Facet Mindfulness Questionnaire; CI, confidence interval. ^∗^*p* < 0.05, ^∗∗^*p* < 0.01, ^∗∗∗^*p* < 0.001.*

With regard to *Dispositional mindfulness measures*, independent Student *t*-tests showed significant differences between groups on MAAS [*t*(58) = -3.463; *p* < 0.01], revealing higher scores for meditators (*M* = 4.31; *SD* = 0.84) compared to non-meditators (*M* = 3.53; *SD* = 0.91). For FFMQ questionnaire, results showed significant differences in all mindfulness facets (all *p*s < 0.05) (see Table 2).

For *anxiety and depression*, no statistically significant differences were observed between groups in HADS scores [*t*(58) = 0.656; *p* = 0.651; *d* = -0.17; 95% CI [-0.66, 0.34]] [(Meditators: *M* = 7.03; *SD* = 3.66) (non-meditators: *M* = 7.70; *SD* = 4.19)]. In relation to *cognitive impairment*, meditators scored significantly lower than non-meditators on the SCIP [*t*(58) = 3.520; *p* < 0.10; *d* = *-*0.90; 95% CI [-1.43, -0.37]].

Results from ANCOVA and MANCOVA controlling by educational attainment yielded statistically significant differences between groups in: *empathy* subscales [*F*(4,53) = 5.790; *p* < 0.01] [*perspective-taking* (*F*(2,57) = 18,326; *p* < 0.001), and *empathic concern* (*F*(2,57) = 13,271; *p* < 0.01)]; *ToM* [*F*(2,57) = 8.597; *p* < 0.01]; *attributional style* [*F*(5,53) = 5.971; *p* < 0.001]; *dispositional mindfulness* (*MAAS*) *F*(2,57) = 13.215; *p* < 0.01, *facets of mindfulness* (FFMQ) [*F*(5,57) = 7.289; *p* < 0.001]; and *cognitive impairment* [*F*(2,57) = 7.973; *p* < 0.01]. The covariate, Educational attainment, was not significantly related to social cognition measures except for *hostility bias* [*F*(1,57) = 12.830; *p* < 0.01] and *aggressivity bias* [*F*(1,57) = 11.525; *p* < 0.01], subscales from *attributional style*.

Pearson correlations analyses demonstrated significant correlations between dispositional mindfulness and social cognition measures for the total sample (see Table 3). Dispositional mindfulness (overall score assessed using the MAAS) was significantly correlated with *empathy* (except for the PD subscale of the IRI) (*r-*values ranging from 0.293 to 0.318), *ToM* (*r* = 0.283; *p* < 0.05) and *attributional style* (only on the aggressivity bias subscale, *r* = -0.380*; p* < 0.01). For mindfulness facets (assessed via the FFMQ), *observing* was significantly correlated with PT (*r* = 0.328; *p* < 0.05) on the IRI, and with the IS (*r* = -0.385; *p* < 0.01), AD (*r* = -0.510; *p* < 0.01), BS (*r* = -0.571; *p* < 0.01), and AB (*r* = -0.391; *p* < 0.01) subscales of the AIHQ. *Awareness* was significantly correlated with *ToM* (*r* = -0.317; *p* < 0.05) and with attributional style [(HB, *r* = 330; *p* < 0.01), (IS, *r* = 0.309; *p* < 0.05), (AS, *r* = 0.345; *p* < 0.01), (BS, *r* = 0.368; *p* < 0.01), (AB, *r* = 0.378; *p* < 0.05)]. The *non-judgment* subscale was significantly correlated with the HB (*r* = 0.369; *p* < 0.01) and AS (*r* = 0.296; *p* < 0.05) aspects of attributional style. For *non-reactivity*, the results revealed a positive significant relationship with *empathy* (FS, PT, and EC subscales with *r-*values between 0.290 and 0.550), and *ToM* (*r* = 0.419; *p* = 0.01), as well as a negative significant correlation with attributional style (for all four AIHQ subscales, *r-*values ranged between -0.556 and -0.289).

**Table 3:** Correlations between mindfulness and social cognition measures for total sample.

	IRI				Eyes Test	HT	AIHQ				
	FS	PT	EC	PD			HB	IS	AS	BS	AB
MAAS	0.293*	0.302*	0.318*	–0.229	0.227	0.283*	–0.380**	–0.145	–0.162	–0.201	–0.212
FFMQ											
Observing	0.183	0.328*	0.201	–0.174	0.089	0.210	–0.199	–0.385**	–0.510**	–0.571**	–0.391**
Describing	0.076	0.217	0.118	–0.146	0.065	0.153	–0.121	–0.094	–0.082	–0.197	–0.049
Awareness	–0.075	–0.143	–0.058	–0.061	–0.112	–0.317*	0.330**	0.309*	0.345**	0.368**	0.378*
Non-judgment	–0.135	–0.218	–0.091	0.132	–0.004	–0.219	0.369**	0.138	0.296*	0.231	0.248
Non-reactivity	0.290*	0.550**	0.381**	–0.172	0.051	0.419**	–0.373**	–0.473**	–0.556**	–0.549**	–0.289*


*IRI, Interpersonal Reactivity Index; FS, fantasy; PT, perspective-taking; EC, empathic concern; PD, personal distress; HT, Hinting Task; AIHQ, Ambiguous Intentions and Hostility Questionnaire; HB, Hostility Bias; IS, Intentionality Bias; AS, Anger Bias; BS, Blame Scale; AB, Aggressivity Bias; MAAS, Mindful Attention Awareness Scale; FFMQ, Five Facet Mindfulness Questionnaire. ^∗^*p* < 0.05, ^∗∗^*p* < 0.01.*

Correlations between mindfulness and social cognition measures for both meditators and non-meditators are include in Table 4.

**Table 4:** Correlations between mindfulness and social cognition measures for meditator and non-meditator samples.

	IRI				Eyes Test	HT	AIHQ				
	FS	PT	EC	PD			HB	IS	AS	BS	AB
**Meditators**											
MAAS	0.030	0.500**	0.306	–0.295	–0.038	0.188	–0.121	0.124	0.134	0.016	0.072
FFMQ											
Observing	0.141	0.354	0.378*	–0.099	0.317	–0.039	0.188	–0.287	–0.274	–0.393*	–0.168
Describing	0.253	0.029	0.122	–0.239	–0.139	–0.055	–0.173	0.068	0.156	–0.087	0.235
Awareness	0.194	–0.215	0.015	–0.309	–0.144	–0.095	–0.088	0.228	0.294	0.297	0.184
Non-judgment	0.031	–0.310	0.074	–0.151	0.067	0.057	0.028	–0.071	0.104	–0.065	–0.088
Non-reactivity	0.473**	0.473**	0.300	0.192	–0.046	0.115	–0.176	–0.362*	–0.377*	–0.419*	–0.038
**Non-meditators**											
MAAS	0.159	–0.157	–0.021	–0.017	0.236	0.194	–0.411*	0.005	–0.034	–0.057	–0.224
FFMQ											
Observing	0.074	–0.216	–0.412*	–0.130	–0.264	0.113	–0.165	–0.086	–0.431*	–0.552**	–0.360
Describing	–0.012	–0.064	–0.204	0.093	0.049	0.138	0.100	0.066	–0.006	–0.037	–0.079
Awareness	–0.090	0.259	0.150	0.175	0.040	–0.364*	0.540**	0.180	0.189	0.256	0.435*
Non-judgment	–0.123	0.191	0.011	0.388*	0.104	–0.234	0.464**	0.054	0.257	0.348	0.321
Non-reactivity	0.309	–0.137	0.031	–0.421*	–0.229	0.385*	–0.251	–0.145	–0.366*	–0.376*	–0.203


*IRI, Interpersonal Reactivity Index; FS, fantasy; PT, perspective-taking; EC, empathic concern; PD, personal distress; HT, Hinting Task; AIHQ, Ambiguous Intentions and Hostility Questionnaire; HB, Hostility Bias; IS, Intentionality Bias; AS, Anger Bias; BS, Blame Scale; AB, Aggressivity Bias; MAAS, Mindful Attention Awareness Scale; FFMQ, Five Facet Mindfulness Questionnaire. ^∗^*p* < 0.05, ^∗∗^*p* < 0.01.*

Stepwise linear regression analysis showed that the *non-reactivity* facet of mindfulness remained the only significant predictor of *empathy* [FS, (β = 0.275; *p* < 0.05; *R*^2^ = 0.08; Δ*R*^2^ = 0.08); PT, (β = 0.541; *p* < 0.001; *R*^2^ = 0.292; Δ*R*^2^ = 0.292); EC, (β = 0.366; *p* < 0.01; *R*^2^ = 0.134; Δ*R*^2^ = 0.134)]; *ToM* (β = 0.41; *p* = 0.001; *R*^2^ = 0.18; Δ*R*^2^ = 0.167); and the *AIHQ-IS* subscale (β = -0.483; *p* < 0.001; *R*^2^ = 0.233; Δ*R*^2^ = 0.233). The PD subscale from IRI was not significantly explained by any of the predictors proposed in the model. For *Emotional recognition*, the only significant predictor that remained in the model was *Educational attainment* (β = 0.366; *p* < 0.01; *R*^2^ = 0.068; Δ*R*^2^ = 0.068)]. In regard to *attributional style*, the HB subscale was significantly explained (*R*^2^ = 0.306; Δ*R*^2^ = 0.068; *F* = 5.369; *p* < 0.05) by *dispositional mindfulness* (MAAS) (β = -0.264; *p* < 0.05), *non-reactivity* (β = -0.301; *p* < 0.05), and *educational attainment* (β = 0.302; *p* < 0.05). For both AS (*R*^2^ = 0.363; Δ*R*^2^ = 0.056; *F* = 4.885; *p* < 0.05) and BS subscales (*R*^2^ = 0.411; Δ*R*^2^ = 0.072; *F* = 6.823; *p* < 0.05), the significant predictors were *non-reactivity* [(AS: β = -0.390; *p* < 0.01), (BS: β = -0.327; *p* < 0.05)] and *observing* [(AS: β = -0.288; *p* < 0.05), (BS: β = -0.395; *p* < 0.01)]. Finally, the AB subscale was significantly explained (*R*^2^ = 0.262; Δ*R*^2^ = 0.107; *F* = 8.128; *p* < 0.01) by *observing* (β = -0.456; *p* < 0.001) and *educational attainment (*β = 0.333; *p* < 0.01).

## Discussion

The present study aimed to explore the role of meditation and dispositional mindfulness on social cognition. The primary aim was to investigate the difference between meditators and non-meditators in social cognition measures. Results were in the anticipated direction and confirmed that meditators performed better on social cognition indices, compared to non-meditators. More specifically, meditators reported comparatively higher scores in *empathy* (expected for the personal distress aspect of empathy), *emotional recognition*, and ToM, and comparatively lower scores in *hostile attributional style/bias*. Findings from the present study are in line with established social cognitive and mindfulness theories, which assert that mindfulness fosters emotional connection to others, mental state attribution, and prosocial behavior (Hutcherson et al., 2008; Birnie et al., 2010; Van Doesum et al., 2013; Tan et al., 2014; Lim et al., 2015). Consequently, the outcomes of this study are inconsistent with those of Melloni et al. (2013), who reported lower levels of empathy in meditators versus non-meditators. Furthermore, our findings also are contrary to Ridderinkhof et al. (2017), who found no significant effect of mindfulness mediation on empathy and mind reading (i.e., ToM).

In addition, significantly higher levels of dispositional mindfulness (assessed by MAAS) were found in meditators compared to non-meditators, which is consistent with the extensive literature supporting the use of meditation to promote mindfulness skills (e.g., Brown and Ryan, 2003; Chambers et al., 2008). In relation to mindfulness facets, meditators reported higher skills of observing, describing, and non-reactivity. However, lower levels of awareness of non-judgment were observed compared to non-meditators. These findings are contrary to those expected and reported by studies indicating increased awareness and non-judgment of inner experience induced by meditation practice (Baer et al., 2006, 2008). We hypothesize that such discrepancies may be due to our sample not being limited to long-term experienced practitioners. Thus, inexperienced meditators may have reported lower levels in these outcomes due to not actually noticing that they were becoming less judgmental.

It is also important to note that when between-group comparisons were controlled by educational attainment, the results remained as they were when initially reported in all dependent variables, except for *emotional recognition*, where no significant differences were found between meditators and non-meditators. Although educational attainment was not a significant covariate for emotional recognition, the regression analyses revealed that it was the only significant predictor in the model. These findings may suggest the implication of educational attainment in emotional recognition, in line with studies suggesting the influence of educational attainment on the neural mechanism of facial expression processing (Demenescu et al., 2014). Moreover, educational attainment was a significant covariate for some facets of attributional style such as hostility bias and aggressivity, which may suggest the potential interference of education on these variables for our sample including both meditators and non-meditators.

A secondary objective of the study was to explore the association between dispositional mindfulness and social cognition. Findings were likewise in the hypothesized direction and are consistent with claims that mindfulness promotes adaptive social functioning (Van Doesum et al., 2013; Melen et al., 2017). More specifically, the results showed that both dispositional mindfulness and mindfulness facets were correlated—to different extents—with social cognition measures for the total sample. Dispositional mindfulness was positively correlated with *empathy* (except for the PD subscale) and *ToM*, negatively correlated with *attributional style* (only on the AB subscale), and was not significantly correlated with *emotional recognition*. However, no significant relationship was observed for the *describing* facet of mindfulness, which suggests that the ability to describe events and personal responses in words (Baer et al., 2006, 2008) is not relevant to social cognition performance. Surprisingly, the *acting with awareness* facet of mindfulness was negatively correlated with *ToM*, and positively correlated with hostile attributional style/bias. There exists evidence suggesting a link between maladaptive forms of awareness (i.e., that presumably relate to practicing aspects of mindfulness incorrectly) and psychopathology, but it is difficult to be certain whether this factor influenced outcomes in the present study (Watkins, 2004; Watkins and Teasdale, 2004; Mehling et al., 2009).

The *non-reactivity* facet of mindfulness showed a stronger correlation with social cognition measures in comparison with other mindfulness facets. This is consistent with mindfulness models that highlight *non-reactivity* as a keystone mechanism of mindfulness (Jimenez et al., 2010). Indeed, it is widely reported that meditation fosters a greater perceptual distance from internal and external cues, which in turn leads to a decentering perspective of experience along with associated reductions in emotional reactivity (Teasdale et al., 2002; Shapiro et al., 2006; Jimenez et al., 2010; Hölzel et al., 2011b; Soler et al., 2014b). According to Lutz et al. (2008), this regulatory role over attention and emotional process enhances the meditator’s social performance skills. Likewise, some researchers advocate a link between meditation and emotional intelligence (Chu, 2010), and we therefore suggest that non-reactivity may play an important role in the application of emotional intelligence during social interactions (Lopes et al., 2004; Brackett et al., 2006).

In terms of the association between dispositional mindfulness and social cognition, our data also revealed different patterns of correlations when taking into account meditation practice, suggesting that dispositional mindfulness in relation to social cognition skills may be influenced by meditation practice. For example, the observing facet was significantly associated with empathetic concern in meditators but inversely so in non-meditators, indicating that paying attention to internal and external experiences such as sensations, thoughts, and emotions decreases empathetic concern in non-meditators. These data support those studies, pointing out that observing is one of the facets most related to and influenced by meditative practice (Lilja et al., 2013; Soler et al., 2014a; Campos et al., 2016). These findings may also suggest that people with no meditation practice may have more difficulty in paying attention to their own internal and external experiences when they have empathetic concerns for others’ sufferings and, therefore, become overwhelmed by the emotions of others. In contrast, for meditators, capacity to observe was associated with empathetic concern, which, according to a number of authors, increases motivation to help others in a selfless attempt to increase their wellbeing (e.g., Batson et al., 1991, 2009). Another interesting result is that dispositional mindfulness (assessed by MAAS) was significantly related to perspective-taking, which has shown to be related to empathetic response and may be an important mechanism in specific meditation practices, together with meta-awareness, cognitive reappraisal, and self-inquiry (Davis, 1983; Batson et al., 2009; Dahl et al., 2015). Contrary to our hypothesis, dispositional mindfulness was not significantly associated with ToM and emotion recognition performance in the meditator sample. Nevertheless, these data are in line with authors who did not find any significant influence of the meditation practice on mind-reading skills (e.g., Tan et al., 2014; Ridderinkhof et al., 2017), and may suggest the implication of other constructs in order to explain the relationship between meditation and both ToM and emotional recognition.

Moreover, our study showed the potential predictors that explain the variance of each social cognition variable. Overall, the non-reactivity facet of mindfulness was the only significant predictor of empathy (fantasy, perspective-taking, and empathetic concern), ToM, and attributional style (intentionality bias). Non-reactivity also significantly predicted *hostility bias* (together with dispositional mindfulness and educational attainment) and both blame and anger bias (together with observing). Observing was also a significant predictor for aggressivity bias (together with educational attainment). In summary, these data highlight non-reactivity as a key facet of mindfulness in order to explain the performance in most of the social cognition measures, and they support the previously mentioned ideas on the importance of non-reactivity as a powerful component of mindfulness outcomes. From a pedagogical perspective, our findings suggest that specific training in mindfulness focused on *observing* internal and external experiences – as well as *non-reactivity* to such inner experience – can result in enhancement of specific social cognition domains (i.e., empathy skills, ToM, and attributional style). Future studies should investigate the mechanisms of mindfulness interventions in respect of their influence on social cognition development.

Collectively, the study findings indicate a significant relationship between dispositional mindfulness and social cognition, and suggest that mindfulness meditation plays a key role in social cognition. More specifically, based on these findings and related studies (Gallagher and Varga, 2015), we suggest the existence of a continuum on social cognition in which healthy meditators and individuals with psychopathology (i.e., schizophrenia, depression or bipolar disorder) are likely to be positioned at opposite poles and where healthy non-meditators are likely to be positioned in the middle section. Our study partially supports this continuum as meditators performed greater on social cognition outcomes versus non-meditators. Nevertheless, further research is needed to support these ideas and to compare differences between meditators, non-meditators and clinical samples. Despite the encouraging findings observed in this study, some limitations should be noted. First, the meditator sample comprised some individuals with only 1 year of meditation experience and, as such, the findings may not be generalizable to individuals with many years or decades of meditation experience. Second, the present study did not assess the type of meditation practiced by each meditator. As a result, the effects of the different meditation practices (e.g., focused attention, open mind, compassion) on social cognition domains were not explored. This is important because different mindfulness practices are known to work in different ways in terms of how they develop mindfulness facets and related meditative competencies (Cebolla et al., 2017). In other words, if mindfulness training were to focus on the facets that are more strongly related to social cognition, it would be reasonable to assume that it would result in a more targeted approach to improving social cognition performance. Third, despite this study employing validated measures with good psychometric properties for the Spanish version, the reliability of some measures was low (α < 0.70) (e.g., Hinting task). Therefore, results pertaining to such measures should be interpreted with additional caution. Another limitation was that the study did not assess all measures that might be relevant to social cognition, such as compassion, self-compassion (Van Dam et al., 2011; García-Campayo et al., 2016; Elices et al., 2017), and emotional intelligence (Chu, 2010). Finally, given that this study followed a cross-sectional design (comparing meditators with non-meditators at one time-point), causal inferences are not possible. Therefore, it is not possible to definitively conclude whether mindfulness actually increases social cognition skills, or whether meditators and non-meditators simply differed in some characteristics initially. For example, perhaps people who choose to engage in mindfulness simply have higher levels of social cognition to begin with, and the reasons for practicing mindfulness meditation could therefore be related to their outcomes (e.g., see Pepping et al., 2016). Further research is needed to address these issues.

To summarize, our findings indicate that meditators perform better in certain aspects of social cognition (i.e., empathy, emotional recognition, ToM, hostile attributional style/bias) in comparison to non-meditators. Moreover, dispositional mindfulness was related to several social cognition outcomes, and mindfulness facets differently predicted social cognition performance, revealing a powerful role for non-reactivity. In the light of these findings, we hypothesize that dispositional mindfulness may enhance performance across several social cognition domains, and that such an enhancement may be promoted by meditation practice. Findings indicating a role for meditation and mindfulness in augmenting social cognition should be researched further in order to better understand this association, as well as how to modify mindfulness-based interventions so that they maximize improvements in social cognitive performance in both clinical and non-clinical study populations. Moreover, findings from social cognition research could be useful in order to conceptualize mindfulness and meditation in a more integrative framework, in line with contemplative cognition approach proposals (e.g., Grossenbacher and Quaglia, 2017). Findings from the present study contribute to understanding the mechanisms related to how we see and navigate to the world and add exploratory data in order to develop future conceptual models explaining the role of meditation and dispositional mindfulness on social cognition domains.

## Ethics Statement

All procedures performed in studies involving human participants were in accordance with the ethical standards of the institutional and/or national research committee and with the 1964 Helsinki Declaration and its later amendments or comparable ethical standards. This study was approved by the Clinical Research Ethics Committee of Aragón (Comité Ético de Investigación Clínica de Aragón, CEICA) which belongs to the Health Research Institute of Aragon (Instituto de Investigación Sanitaria de Aragón, IIS Aragón), from Zaragoza (Spain). Informed consent was obtained both informed and written by all individual participants included in the study.

## Author Contributions

DC analyzed the data and wrote the manuscript. MM-A and MN-G collaborated in the writing and editing of the final manuscript. YL-d-H designed the study, and collaborated in the writing of the study and editing of the final manuscript. MG-P designed and executed the study, and collaborated in the writing and editing of the final manuscript. WVG and ES collaborated in the writing and editing of the final manuscript. JG-C designed the study, assisted with the data analyses, and collaborated in the writing and editing of the final manuscript.

## Conflict of Interest Statement

The authors declare that the research was conducted in the absence of any commercial or financial relationships that could be construed as a potential conflict of interest.
